# Nutritional Needs, Dietary Knowledge, and Culinary Skills in Individuals with Diabetes Mellitus: A Scoping Review

**DOI:** 10.1016/j.advnut.2026.100627

**Published:** 2026-04-07

**Authors:** Jorge Casaña Mohedo, María Teresa Murillo-Llorente, Alma Palau Ferre, María Faus, Barbara Gómez Taylor, Francisca Esteve Claramunt, Sara Morales Palomares, Elena Sandri

**Affiliations:** 1 Sobrepeso, Obesidad, Nutricion y Estilos de Vida Research Group, Faculty of Medicine and Health Sciences, Catholic University of Valencia San Vicente Mártir, Valencia, Spain; 2 Department of Nursing, Catholic University of Valencia San Vicente Mártir, Valencia, Spain; 3 Faculty of Medicine and Health Sciences, Catholic University of Valencia San Vicente Mártir, Valencia, Spain; 4 Department of Nutrition and Dietetics, Catholic University of Valencia San Vicente Mártir, Valencia, Spain; 5 Department of Nursing, Faculty of Nursing and Chiropody, Universitat de Valencia, Valencia, Spain; 6 Department of Pharmacy, Health and Nutritional Sciences, University of Calabria, Rende, Italy

**Keywords:** diabetes mellitus, culinary medicine, cooking skills, nutritional requirements, scoping review

## Abstract

Dietary management is a cornerstone of diabetes mellitus (DM) care. However, a persistent disconnect remains between theoretical nutritional knowledge, what individuals are advised to eat, and the practical culinary skills required to translate these recommendations into daily practice. This gap is frequently compounded by socioeconomic constraints and cultural determinants. To map the available evidence on dietary knowledge, culinary skills, and food-related practices among people with DM, with particular attention to barriers and facilitators in daily-life settings. A scoping review was conducted following the Joanna Briggs Institute methodology and Preferred Reporting Items for Systematic Reviews and Meta-Analyses for scoping reviews guidelines. Major databases (MEDLINE/PubMed, Scopus, Web of Science, and Embase) were searched for studies published between 2014 and 2025. Extracted variables were mapped deductively to the World Health Organization International Classification of Functioning, Disability and Health, distinguishing self-care and dietary management (d5) from domestic-life competencies related to meal preparation (d6). Forty-six studies were included. The evidence base was heterogeneous and focused largely on dietary self-management, whereas comparatively few studies explicitly assessed instrumental cooking skills (*n* = 8). Across studies, structural barriers, particularly food insecurity and the cost and availability of healthier foods, frequently limited the feasibility of recommended diets. Skills-based interventions, including culinary medicine and hands-on cooking programs, were associated with improvements in culinary self-efficacy and, in some studies, clinically meaningful hemoglobin A1c reductions. Family dynamics, peer support, and culturally shaped practices (including religious observances) emerged as important determinants of adherence. Current diabetes education strategies risk achieving conceptual adequacy without practical applicability. Effective dietary management in DM requires a shift from predominantly prescriptive nutritional advice toward skills-based approaches grounded in culinary medicine. Future interventions should be culturally responsive and explicitly address environmental and social barriers to bridge the gap between clinical recommendations and real-world implementation.

This systematic review followed a protocol registered prospectively on Open Science Framework available at: https://doi.org/10.17605/OSF.IO/J9PHQ.

**Statement of significance**

This scoping review uniquely uses the WHO International Classification of Functioning, Disability and Health framework to disentangle what diabetes education asks patients to know and manage (d1/d5) from what they must be able to do in daily life, meal preparation, and resource management (d6), and from the interpersonal determinants of eating (d7). Mapping 46 studies (2014–2025) reveals a clear imbalance toward knowledge/adherence outcomes, with comparatively little measurement of cooking skills and limited incorporation of social support in digital solutions, pointing to an actionable shift toward skills-based culinary medicine.

## Introduction

Diabetes mellitus (DM) is a chronic metabolic disorder characterized by persistent hyperglycemia due to defects in insulin secretion, insulin action, or both, and it represents one of the fastest-growing public health challenges worldwide [1,2]. Over the last 3 decades, the global incidence and prevalence of DM have risen steadily, with Scientific Reports analyses based on Global Burden of Disease data documenting sustained increases in incidence, mortality dynamics, and mortality-to-incidence ratios across regions [3]. Cardiovascular and renal complications account for much of the morbidity and mortality burden, with cardiovascular disease remaining the leading cause of death, and diabetic kidney disease mortality increasing in many settings [[4], [5], [6]].

Medical nutrition therapy is a cornerstone of diabetes care because dietary choices influence glycemic control and cardiometabolic risk factors, with downstream implications for long-term complications [7]. Evidence from systematic reviews and meta-analyses indicates that low glycemic index (GI)/glycemic load (GL) dietary patterns can modestly reduce hemoglobin A1c (HbA1c) and improve cardiometabolic risk factors beyond pharmacotherapy in adults with type 1 and type 2 diabetes (T1D and T2D) [8]. Other syntheses suggest that carbohydrate-restricted approaches and several dietary patterns (Mediterranean, low-GI/GL, vegetarian/vegan, higher protein) can achieve clinically relevant improvements in glycemic and cardiometabolic outcomes [[9], [10], [11]]. Nutrition interventions delivered within diabetes self-management education and support (DSMES) programs are associated with HbA1c reductions and improved diet quality [12].

Importantly, patients’ ability to translate “what to eat” into everyday practice, both in diabetes and in the general population, depends on their level of food literacy, which encompasses nutrition knowledge, culinary skills, practical competencies, and food-related behaviors [12,13,14]. In widely adopted definitions (including that proposed by the FAO), food literacy encompasses knowledge, skills, competencies, and behaviors related to what, how, when, and how much to eat, tailored to context and health needs while considering sustainability and the social dimension of food [15]. Culinary medicine and cooking-skills interventions aim to bridge this implementation gap by integrating hands-on cooking education with nutrition guidance to strengthen food literacy, dietary self-efficacy, and the long-term adoption of healthy dietary patterns [16].

In adults with T2D, skills-based programs appear promising. A randomized trial of a teaching-kitchen curriculum showed feasibility with improvements in blood pressure and cholesterol and favorable trends in HbA1c [17]. A randomized 6-wk intervention combining cooking and DSMES also improved dietary behaviors, vegetable intake, and psychosocial outcomes, with notable benefits among participants experiencing food insecurity [12].

Nevertheless, evidence mapping highlights heterogeneity in delivery models and outcomes, and inconsistent effects on hard cardiometabolic endpoints [16]. Social determinants of health, such as food insecurity, community resources, and home/work environments, strongly shape food choices and diabetes self-management [18,19]. Food insecurity can drive coping strategies (e.g., reliance on low-cost energy-dense foods) that undermine glycemic outcomes [20], whereas rural and resource-limited contexts create access barriers to traditional in-person education [21,22], motivating culturally adapted and distance-learning cooking interventions [23].

Given the breadth and heterogeneity of this emerging evidence base, spanning different populations, settings, and outcomes, a scoping review is warranted to comprehensively map what is known about nutritional needs, dietary knowledge, culinary skills, and usual food-related practices among people with DM [16,23]. To clarify conceptual distinctions that matter for both research and practice amidst this heterogeneity, we adopt the WHO’s International Classification of Functioning, Disability and Health (ICF) as an organizing framework [24]. The ICF differentiates body functions from activities and participation and recognizes environmental and personal factors that modulate functional performance. Applied to diabetes-related nutrition, this perspective helps distinguish: *1*) metabolic functions that motivate nutritional prescriptions, *2*) diet-management processes required to interpret and follow those prescriptions, and *3*) instrumental meal-preparation abilities needed to enact dietary plans in everyday life. This framing foregrounds a common but underappreciated gap: evidence describing what patients should eat is abundant, yet fewer studies evaluate whether individuals possess the practical cooking skills and domestic resources necessary to operationalize those recommendations. Likewise, environmental determinants (e.g., food access, affordability, family support, and kitchen infrastructure) interact with personal capacities to shape dietary behavior. Therefore, the objective of this scoping review was to map the literature on nutritional needs and metabolic targets, dietary knowledge and diet-management skills, culinary competencies and meal-preparation practices, and the contextual factors that facilitate or hinder implementation across real-world settings.

## Methods

### Study design

This scoping review was conducted following the methodological framework proposed by Arksey and O’Malley [25] and later refined by the Joanna Briggs Institute (JBI) [26]. Reporting was guided by the PRISMA for scoping reviews (PRISMA-ScR) [27].

In line with Munn et al. [28], a scoping approach was chosen rather than a systematic review because the aim was to map the available literature on the nutritional needs and culinary–nutrition competencies of people with diabetes. Specifically, we sought to identify nutritional needs, explore dietary and culinary knowledge as well as cooking skills, and describe eating habits and usual food-related practices in this population.

### Institutional review board statement

This study was exempted from ethical review and approval because it is a review of existing literature and was not conducted on humans or animals.

### Protocol and registration

The review protocol was developed a priori and registered on the Open Science Framework under the identifier https://doi.org/10.17605/OSF.IO/J9PHQ, as recommended to ensure transparency of the process.

### Eligibility criteria

Inclusion and exclusion criteria were defined using the Population, Concept, Context mnemonic (PCC) recommended by JBI methodology [26]:

• Population: studies involving individuals with T1D and T2D, with no restrictions by age, sex, or ethnicity.
• Concept: the main focus was on nutritional needs, culinary knowledge, cooking skills, and eating habits.
• Context: studies conducted in hospital, outpatient/ambulatory, community, and/or home settings were considered.
• Types of sources: we included peer-reviewed original articles, systematic reviews and other literature reviews, clinical guidelines, educational programs, and grey literature. No language restrictions were applied; however, the search was limited to studies published between 2014 and 2025. For studies published in languages other than English, titles and abstracts were screened using translation tools and dictionaries; however, all articles that ultimately met the final inclusion criteria were published in English.

### Exclusion criteria

Articles were excluded if they did not meet the PCC eligibility criteria defined above or if they did not provide data relevant to the review objectives. Specifically, we applied the following exclusion criteria:

• Population: animal or *in vitro* studies were excluded, as were studies focused exclusively on metabolic conditions other than DM (e.g., diabetes insipidus or obesity without confirmed comorbid DM).
• Concept: we excluded studies limited to pharmacological interventions, bariatric surgery, or artificial nutrition (enteral/parenteral) when these did not include an educational component, oral dietary self-management, or an assessment of cooking-related skills. We also excluded manuscripts that did not report extractable data on food-related knowledge, attitudes, or practices.
• Publication type: conference abstracts, letters to the editor, and editorials without original empirical data or substantial theoretical synthesis were excluded, as were full texts that could not be retrieved after contacting the authors.

### Information sources and search strategy

A comprehensive search was conducted in 4 major bibliographic databases with broad coverage in health sciences: MEDLINE (via PubMed), Scopus, Web of Science, and Embase. This combination was selected to ensure high sensitivity for clinical and biomedical literature, as well as wide coverage of nutrition and dietetics journals. To reduce publication bias and capture nonindexed evidence, we also performed a supplementary search in the Mendeley collaborative catalog and conducted manual reference checking (“snowballing”) of the studies included in the review.

The search strategy was developed in 3 steps, following JBI recommendations [26]:

1. An initial, limited search in MEDLINE to identify relevant English keywords and medical subject heading terms (Table 1).

**TABLE 1:** Keywords and health descriptors used in the search

Keywords	DeCS term (English)	Descriptor ID
Diabetes	Diabetes mellitus	D003920
Nutrition	Diet, Food and Nutrition	D000066888
Nutrition	Nutrition programs	DDCS016570
Eating habits	Feeding behavior	D005247
Cooking	Cooking	D003296
Culinary skills	Free-text term	

Abbreviation: DeCS, Health Sciences Descriptors.
2. A full search using all identified keywords and index terms across all included databases (Supplemental Table 1).
3. All identified records were exported to Mendeley strictly for reference management and deduplication. The subsequent blinded screening process (title/abstract and full-text) was conducted independently by 2 reviewers using a dedicated, formatted Microsoft Excel spreadsheet designed for systematic reviews. (see Supplemental Table 1 for the full strategy.)

### Study selection

**FIGURE 1:**
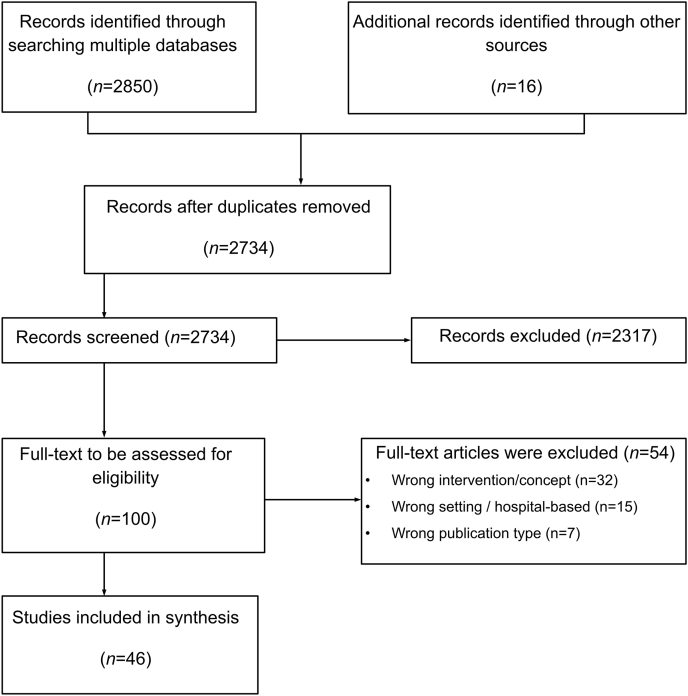
PRISMA flowchart.

All records identified were imported into Mendeley strictly for reference management and deduplication. Following this step, the blinded screening process (title/abstract and full-text) was conducted independently by 2 reviewers (JCM and ES) using a dedicated, formatted Microsoft Excel spreadsheet. The reviewers then compared their inclusion decisions, and any disagreements were resolved by consensus with a third reviewer (MTM-L). A PRISMA flow diagram (Figure 1) was used to document the selection process and reasons for exclusion.

### Data extraction

Data were extracted using a standardized Excel form that was pilot-tested by the research team. Two reviewers independently extracted data to ensure accuracy (JCM and ES). The extracted variables included:

• Study characteristics (author, year, country, and design).
• Population (sample size, age, and diagnosis).
• Intervention/concept details (e.g., type of technology, measurement instrument).
• Key findings relevant to the review question.
• Reported barriers and facilitators identified across the included studies.

Study-level extracted data are reported in Supplemental Table 2.

### Data synthesis

Extracted data are presented through a narrative synthesis supported by tables and visual evidence maps.

To ensure international standardization of findings and to facilitate comparability, variables related to culinary competencies and self-care were deductively mapped using the WHO’s ICF framework [24]. Specifically, we used codes from the Domestic Life domain (d6) to classify culinary skills and from the Self-care domain (d5) to classify eating habits, in line with the ICF Core Set for DM.

## Results

### Study selection

The database search yielded a total of 2866 records. After removing duplicates, 2734 records were screened by title and abstract. Of these, 100 articles were assessed in full text for eligibility. Ultimately, 46 studies were included in the qualitative synthesis. The study selection process is shown in the PRISMA-ScR flow diagram [29] (Figure 1).

### General characteristics of the evidence

**FIGURE 2:**
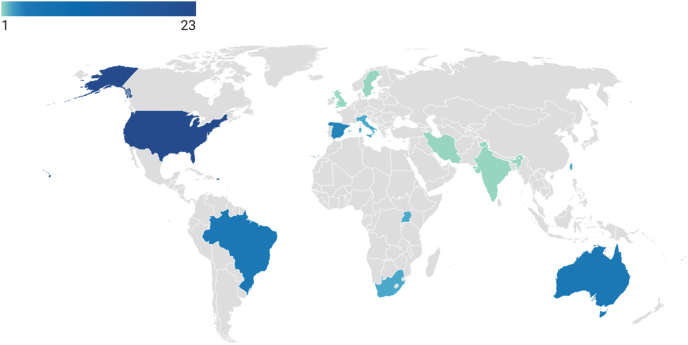
Geographic distribution of included studies (choropleth map).

Regarding geographic distribution, the included studies were predominantly conducted in the United States (*n* = 23; 50%), followed by a notable contribution from emerging economies such as Brazil (*n* = 4) [[30], [31], [32], [33]] and African countries (Uganda and South Africa, *n* = 4) [[34], [35], [36], [37]]. The remaining studies (*n* = 15) were primarily distributed across European countries (*n* = 7) and Asian countries (*n* = 4), with a few additional studies from other regions (Figure 2).

In terms of study design, the evidence base was highly heterogeneous. Qualitative studies were the most frequent (*n* = 13), followed by experimental studies, including randomized controlled trials (RCTs) and pilot studies (*n* = 14), primarily evaluating culinary medicine interventions. The remaining records comprised other observational designs, as well as systematic and narrative reviews, included to capture current trends in the field.

Sample sizes varied widely, ranging from in-depth qualitative studies with as few as 7 participants [38] to population-based database analyses including >16,000 records [39]. Most studies focused on adults with type 2 diabetes, with a predominant age range between 50 and 70 y. However, a meaningful subgroup of studies (*n* = 2) explicitly targeted children, adolescents, or young adults with T1D and their families, underscoring the potential value of early culinary education.

Regarding context, and in line with the eligibility criteria, most investigations moved beyond traditional clinical settings and were conducted in community and home-based environments (e.g., food pantries, community centers, and participants’ own homes). Interventions largely emphasized hands-on cooking and skills-based education. A recent increase was observed in virtual and telehealth formats following the pandemic, as well as in food prescription (“*Food as Medicine*”) programs implemented among populations experiencing food insecurity (Table 2).

**TABLE 2:** General characteristics and findings grouped by type of intervention

Intervention type	Included studies (references)	Population and estimated total sample	Key characteristics and main group-level findings
Practical culinary education (“hands-on”/teaching kitchens)	Williams et al. (2021) [12]; Byrne et al. (2017) [40]; Dexter et al. (2019) [41]; Shrodes et al. (2021) [42]; Aparecida et al. (2020) [30]; Venkatesh et al. (2024) [43]; Goldstein et al. (2024) [44]; Sharma et al. (2021) [45]	*n* >2000. Mainly T2D, veterans, and food-insecure populations.	• Programs based on participatory cooking workshops. • Outcome: consistent improvements in culinary self-efficacy and reductions in HbA1c and blood pressure. • More effective than standard education in changing home-based habits.
Family-based and culturally adapted interventions	Baig et al. (2015) [46]; Burner et al. (2018) [47]; Hempler et al. (2015) [48]; Murillo et al. (2022) [49]; Quiñonez et al. (2014) [38]; Shapiro et al. (2024) [50]; Tripathi et al. (2023) [51]; Muchiri et al. (2021, 2023) [35,36]; Kiguli et al. (2019) [34]	*n* ≈ 400 + reviews. Immigrants, ethnic minority groups, and families.	• Qualitative studies and reviews focused on the social environment. • Outcome: identification of “family sabotage” and cultural conflict as major barriers. • Cultural adaptation of recipes emerged as a key facilitator.
Food literacy and theory-based education	Savarese et al. (2021) [52]; Bastami et al. (2023) [53]; Uliana et al. (2022) [32]; Stotz et al. (2021) [54]; Panduro et al. (2024) [55]; Araújo et al. (2023) [31]; Bowen et al. (2015) [56]; Daramilas et al. (2025) [57]; Vasconcelos et al. (2021) [58]	*n* >1000. T1D and T2D (often in clinical-oriented settings).	• Focus on knowledge: label reading, carbohydrate counting, concept maps. • Outcome: food literacy was directly associated with glycemic control. • Limitation: theoretical knowledge does not consistently translate into practice without skills training.
Innovation, technology, and food provision	Misra et al. (2025) [59]; Rayala et al. (2025) [60]; Short et al. (2022, 2023, 2025) [[61], [62], [63]]; Biber (2023) [64]; Hawley et al. (2021) [23]; Lai et al. (2023) [65]	*n* >17,000 (including large databases and pilot studies).	• Use of apps, “food pharmacy”/food boxes, and telemedicine. • Outcome: food provision helps remove financial barriers. • Apps can support tracking, but may lead to long-term fatigue and disengagement.
Cross-sectional studies of behaviors and habits	Weller et al. (2021) [66]; Sińska et al. (2022) [67]; Wetherill et al. (2019) [39]; Hung et al. (2022) [68]; Vasconcelos et al. (2021) [58]; Addala et al. (2019) [69]; Almansour et al. (2016) [70]	*n* >18,000. Population surveys and observational studies.	• Analysis of real-world dietary patterns. • Outcome: identification of structural barriers (time, work demands, cost). • Consistently suggests that home cooking (compared with eating out) is protective.

Abbreviations: HbA1c, hemoglobin A1c; T1D, type 1 diabetes; T2D, type 2 diabetes.

### Thematic mapping using the WHO ICF framework

Although community- and home-based studies predominated, eligible evidence also encompassed clinical settings, including hospital-based and outpatient/ambulatory services. Accordingly, when evidence was classified using the WHO ICF, community-based literature was not confined to traditional Self-care (d5) but predominantly emphasized the Domestic Life domain (d6).

This pattern suggests that when education moves beyond the hospital setting, interventions tend to shift from information delivery toward practical training in functional, everyday skills.

Moreover, by focusing on daily life, the review also highlights the importance of interpersonal interactions and relationships (d7). Unlike clinical settings, where the relationship is primarily clinician–patient, diabetes management in the home often becomes a more collective experience, shaped by family dynamics and cultural context (Table 3).

**TABLE 3:** Distribution of studies according to ICF domains

ICF code	Domain/concept	Description	Studies (*n*)
d1 (d155, d166 and d175)	Learning and application of knowledge	It focuses on Food Literacy, the acquisition of cognitive (nonmanual) skills such as carbohydrate counting, reading labels, understanding concept maps, and validating educational materials.	8
d5 (d550 and d570)	Self-care	It addresses direct eating behavior (act of eating), adherence to prescribed dietary guidelines, habit recording, weight management, and adapting the diet to everyday situations without an explicit cooking component.	10
d6 (d630 and d610)	Domestic life	It encompasses culinary skills practices, preparing meals at home, planning menus, buying food, and managing resources in contexts of food insecurity.	17
d7 (d760 and d710)	Interpersonal interactions and relationships	It analyzes the influence of the social environment on food: support vs. family sabotage, cultural and religious barriers, group dynamics in educational workshops and the relationship with the community.	9

Abbreviation: ICF, International Classification of Functioning, Disability and Health.

**FIGURE 3:**
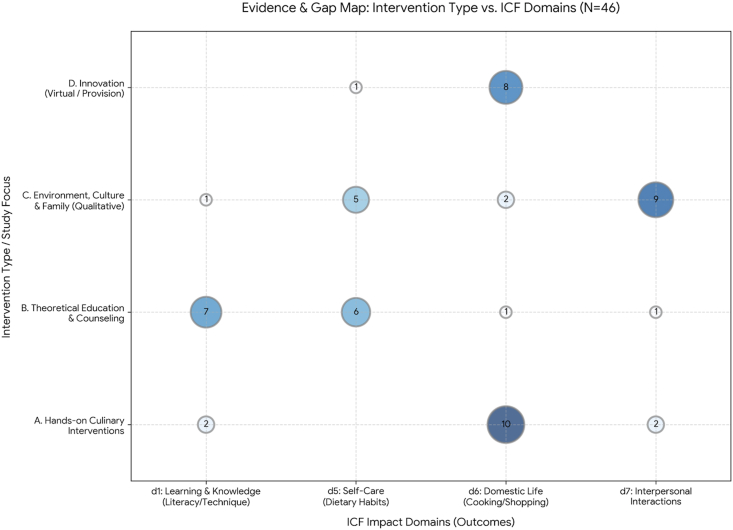
Evidence and gap map of included studies by intervention type and ICF impact domains. Rows represent intervention type (A: hands-on culinary interventions; B: theoretical education and counseling; C: environment, culture, and family—qualitative; D: innovation, including virtual delivery and/or food provision). Columns represent ICF domains (d1: learning and knowledge; d5: self-care; d6: domestic life; d7: interpersonal interactions). Bubble area is proportional to the number of studies in each cell; numbers inside bubbles indicate the study count (*n*). Darker shading denotes higher counts. Empty cells indicate evidence gaps (*n* = 0). Note: The total count of studies represented in the bubbles (*n* = 55) exceeds the total number of included articles (*n* = 46) because complex interventions often addressed multiple ICF domains simultaneously and were therefore mapped to more than one intersection. ICF, International Classification of Functioning, Disability and Health.

Once the evidence map was developed (Figure 3), a synthesis tool that organizes and visualizes the available studies by intervention type and by the impact domains of the ICF, a clear pattern emerged in the distribution of existing knowledge. In particular, there was a strong concentration of evidence at the intersection of practical (“hands-on”) interventions and the Domestic Life domain (d630; *n* = 10). This finding supports the idea that, for effective diabetes management in the home setting, theoretical knowledge transfer alone (row B) is not sufficient. Direct instrumental practice, such as cooking workshops, is essential to help individuals translate dietary recommendations into concrete actions in everyday life.

On the other hand, the evidence map suggests a disconnect between qualitative and intervention research. A marked clustering of studies links interpersonal relationships (d7) with qualitative designs; however, the same domain is largely absent in studies focused on innovation (row D). This indicates that family and social support, often highlighted as central to diabetes self-management, has been underutilized in the design of technology-based interventions and educational innovations.

In the same area (row D), most studies cluster within d6 (Domestic Life). This may indicate that technology is primarily being used to address logistical challenges, such as access to food, recipes, or shopping lists, whereas it has not yet been leveraged to support interpersonal relationships and social support in diabetes care.

Therefore, the evidence suggests a gap in the design of technology-based interventions that strengthen the social dimension of diabetes self-management, as most innovations continue to focus on the individual rather than on shared or family-supported practices.

#### Learning and applying knowledge (d155, d166, and d175)

The d1 domain includes studies assessing individuals’ ability to process complex information and develop specific cognitive skills. In the included evidence (*n* = 8), this domain was addressed across 3 main areas: food literacy, technical/mathematical competence, and validation of educational tools.

First, the literature highlights food literacy [52,53] as an important clinical predictor, showing that the ability to read, understand, and use nutrition information (d166) is inversely associated with glycated hemoglobin levels. At the same time, several studies suggest that providing information alone is insufficient when cognitive barriers, such as low educational attainment or difficulty interpreting labels, are not explicitly addressed.

At a more instrumental level, related to skill acquisition (d155) and problem-solving (d175), interventions focusing on calculation and estimation were prominent. Diabetes self-management often requires advanced numeracy for carbohydrate counting [32] and visuospatial skills for portion-size estimation [37,55]. Overall, the findings suggest that errors in these tasks are more often linked to limited training in these specific skills than to a lack of motivation.

Finally, a subgroup of methodological studies [31,71] emphasizes that effective learning depends on protocols that are expert-validated and culturally adapted, ensuring that individuals do not merely receive information but develop meaningful cognitive self-efficacy before translating knowledge into action.

#### Self-care and health management (d550 and d570)

The d5 domain encompasses behaviors related to managing one’s own health (d570) and the act of eating (d550), understood as part of an ongoing behavioral pattern. Studies classified in this category (*n* = 10) moved away from hands-on culinary interventions and instead focused on behavioral adherence, coping strategies, and the adaptation of dietary management to complex real-life contexts.

A key theme was the tension between clinical rigidity and everyday flexibility. Weller and Vickers [66] and Lai et al. [65] suggest that successful self-care is less about following a “perfect” restrictive diet and more about developing sustainable lifestyle strategies that individuals can maintain without burnout.

Dietary tracking was also described as a double-edged tool [59]. It may increase awareness and improve short-term behaviors, but it can lead to “tracking fatigue,” which undermines longer-term adherence.

This analysis further indicates that self-care is highly situational. As noted by Almansour [70], diabetes management (d570) often needs to be renegotiated during religious observances such as Ramadan, when eating schedules (d550) change substantially. This highlights that self-care competence also involves adapting medical guidance to the patient’s cultural and temporal realities.

Finally, several authors [12,33,69] emphasize that, particularly among young adults and vulnerable populations, glycemic control may compete with other self-care priorities, such as weight management or stress coping. When these issues are not addressed holistically, they may contribute to disordered or inconsistent eating behaviors.

#### Domestic life: culinary skills and resource management (d610 and d630)

The d6 domain represents the core of this review and includes the largest body of evidence (*n* = 17). This section examines individuals’ ability to carry out complex household tasks, specifically meal preparation (d630) and acquiring goods and services (d610). Unlike traditional education models, interventions in this domain focus less on memorizing dietary rules and more on developing instrumental know-how and practical logistical competence.

The most consistent finding was the superiority of hands-on approaches. Across clinical trials and pilot studies [40,42,49], teaching-kitchen interventions were more effective than theory-based education in improving culinary self-efficacy. Evidence suggests that when participants actively handle foods and learn basic cutting skills and healthy cooking techniques, anxiety around meal preparation decreases and home cooking becomes more frequent, an important behavior that is inversely associated with metabolic risk [68,72].

This domain also captures resource management (d610) in vulnerable contexts. The review identifies a critical line of research on food insecurity [39,61], where cooking education is paired with direct food provision. These studies converge on a practical point: cooking skills alone have limited value if individuals lack the means or access to obtain recommended ingredients.

Finally, technological innovation has enabled these interventions to be delivered directly to participants’ homes. Virtual programs [44,45] indicate that “tele-cooking” is not only feasible but may also support skill transfer, as participants practice in their own kitchens with their own equipment, reducing the gap between training settings and real-world domestic routines.

#### Interpersonal interactions and relationships (d710 and d760)

Studies mapped to this domain (*n* = 9), predominantly qualitative, examined how family relationships (d760) and adherence to cultural and community norms shape individuals’ ability to follow dietary recommendations.

The most recurrent finding was the dual role of the family as both a barrier and a facilitator. Systematic reviews and qualitative studies [46,51,54] describe “family sabotage” (unintentional or explicit), where pressure to consume unhealthy foods during social gatherings undermines patients’ efforts. Conversely, when interventions involve the family as a unit of care [47], the home environment can become a key driver of adherence.

Cultural competence also emerged as a critical factor. Several studies [48,50] document the internal conflict experienced when medical recommendations clash with identity-related or religious values. In many cultures, refusing food may be perceived as disrespectful or a violation of hospitality norms (d710), prompting individuals to prioritize social etiquette over metabolic health. For example, among Indigenous communities, food can represent a sacred connection to land and community; interventions that overlook this symbolic meaning are unlikely to succeed [38].

Finally, peer socialization appeared to have therapeutic value. Studies of group-based workshops [30,73] indicate that learning to cook in a group can create a psychologically safe environment. Sharing experiences with peers (“vicarious learning”) may reduce isolation and normalize living with diabetes, benefits that are difficult to achieve through 1-to-1 clinical education alone.

### Barriers and facilitators for culinary and nutrition education

This analysis identified a set of key determinants that modulate adherence and the overall success of interventions (Table 4).

**TABLE 4:** Synthesis of barriers and facilitators

Factor level	Barriers (hindering factors)	Facilitators (enabling factors)
Individual (cognitive and behavioral)	• Low literacy/numeracy: difficulty counting carbs or reading labels [32,56]. • Time constraints: perception that healthy cooking is time-consuming [68,72]. • Digital fatigue: burnout from logging data in Apps/Tracking fatigue [59]. • Cooking anxiety: lack of confidence in basic culinary skills [41].	• Culinary self-efficacy: feeling “capable” after hands-on workshops [12,44]. • Visual learning: use of maps, visual portions, and diagrams [55,57]. • Flexibility: adapting diet to lifestyle rather than rigid rules [66].
Social/cultural (environment and interactions)	• Family sabotage: relatives bringing unhealthy food home or undermining efforts [46,51]. • Social obligation: pressure to eat during hospitality events or festivities [48,70]. • Cultural identity conflict: feeling that the medical diet erases cultural heritage [38].	• Peer support: group learning and socialization with other patients [30,73]. • Family involvement: engaging family members in shopping and cooking [47]. • Cultural competence: providers respecting and adapting traditional recipes [36,50].
Structural (resources and access)	• Food insecurity: poverty and lack of access to fresh produce [39,61]. • Cost: perception that healthy food is too expensive [51]. • Digital divide: lack of internet access or devices for telemedicine [45].	• Food provision: programs providing free ingredients (Food Rx) alongside education [61,64]. • Budgeting skills: teaching how to shop on a budget [23,60]. • Workplace environment: facilities to cook or eat healthy meals at work [68].

Food insecurity and the high cost of fresh foods stood out as the main structural barriers [34,39]. Several studies point out that cooking education has little impact when people simply cannot afford or access the ingredients being recommended. At the individual level, lack of time, often linked to long working hours, and decision fatigue, including “tracking fatigue” in digital interventions, were frequently reported [59]. From a cultural standpoint, clashes between biomedical advice and hospitality norms or religious traditions can create value-based tensions that commonly lead to reduced adherence or abandonment of dietary changes [48,69].

Among facilitators, experiential (“hands-on”) learning was consistently the most prominent. Actively cooking and practicing techniques tended to build self-efficacy far more than receiving information in a passive way [12,40]. At the social level, peer support in group formats and meaningful family involvement often shifted the home environment from a context of “sabotage” to one of support [47,73]. Finally, culturally tailoring recipes and using visual tools (e.g., maps and videos) appeared to provide crucial cognitive scaffolding for people with lower literacy levels [55,57].

## Discussion

This scoping review synthesizes evidence from 46 studies and highlights that diabetes management is increasingly enacted in domestic and community environments rather than exclusively within clinical settings. Using the ICF enabled a structured interpretation of this literature and brought into focus a recurring imbalance: research and education have largely concentrated on prescriptive self-care (d5), whereas the instrumental competencies needed to operationalize dietary recommendations in everyday cooking and household routines (d6) and the social-relational context shaping eating practices (d7) are comparatively less addressed.

### Main findings under the ICF framework

Across the included evidence, there is a clear shift toward home- and community-based contexts, with substantial attention to the Domestic Life domain (d6) rather than body functions alone. This pattern aligns with the notion that glycemic control is largely determined by daily routines and food-related practices in real life, not only by clinical encounters [30,69]. At the same time, the evidence remains fragmented. Food literacy is consistently recognized as clinically relevant [57,66], yet many interventions emphasize knowledge and dietary planning whereas implicitly assuming that education will translate into practical competence. Our mapping suggests that this assumption may be problematic when culinary confidence, meal-preparation skills, and household resources are not assessed or supported.

### Conceptual contribution of this scoping review

Beyond mapping existing interventions, this scoping review makes a conceptual contribution by operationalizing dietary self-management in diabetes as a set of distinct functional domains rather than a unitary behavior. By applying the ICF framework, we demonstrate that “dietary adherence” conflates ≥3 separable capacities: cognitive understanding (d1), behavioral regulation (d5), and instrumental domestic competence (d6). This distinction helps explain why traditional education-centered interventions often fail despite high levels of nutritional knowledge. To our knowledge, this is the first review to systematically differentiate these domains in the context of diabetes nutrition and to position culinary skills (d630) as a measurable functional outcome rather than a peripheral educational add-on.

### From “what to eat” to “how to cook”: the educational paradox (d5 compared with d6)

A central theme emerging from the mapped evidence is the tension between the demands of self-care (d570) and limitations in instrumental capacity (d630). Individuals are often expected to adopt complex behaviors, such as carbohydrate counting or dietary tracking, without parallel training or support for practical meal preparation and routine management. Misra et al. [59], for example, describe “tracking fatigue” in mobile interventions: individuals recognize the need to record meals (d5), yet the sustained cognitive burden in the absence of efficient planning and household routines (d6) can drive disengagement. Similarly, the mathematical demands of carbohydrate counting represent a technical barrier that may affect diet quality and feasibility [32,74].

In contrast, the evidence unequivocally favors hands-on interventions. Culinary Medicine programs and practical workshops have been shown to be superior to standard education, achieving not only improvements in self-efficacy but also tangible reductions in HbA1c and blood pressure [17,69]. This confirms that transforming an abstract recommendation into a manual skill, such as cutting techniques or batch cooking, reduces anxiety associated with food preparation and improves long-term adherence [40,75,76]. Despite this robust evidence, hands-on interventions have not been widely adopted in clinical practice. This gap may stem from a systemic lack of emphasis on prevention within many medical constructs and a “system-wide” lack of incentive to prioritize lifestyle changes over pharmacological intensification, often driven by profit motivations. Furthermore, the high-touch nature of these interventions, requiring specialized kitchens and trained staff, presents significant logistical and financial challenges compared with traditional counseling.

Major international diabetes guidelines emphasize individualized nutrition therapy and structured self-management education, but frequently frame dietary management primarily as a cognitive and behavioral process. Although social determinants are increasingly acknowledged, explicit assessment and training of instrumental culinary skills are rarely foregrounded as core components of routine care. Our synthesis suggests that this may represent a practical blind spot: without attention to domestic food preparation capacity (d630), guideline-consistent advice can remain theoretically sound yet difficult to implement for individuals with low food literacy, limited time, or constrained resources.

### Social and environmental determinants: connecting “d” and “e”

Analysis using the ICF framework reveals that execution capacity (d domains) is intrinsically dependent on Environmental Factors (e chapter). Food insecurity (e570) emerges as the most severe structural determinant. Studies such as those by Wetherill et al. [39] and Sharma et al. [45] demonstrate that, in the absence of economic resources, patients adopt negative coping strategies, such as diluting food or resorting to ultraprocessed products, regardless of their level of nutritional knowledge.

Across settings, the evidence also points to what can be interpreted as culinary skill erosion, a progressive loss of confidence, autonomy, and competence in home cooking driven by time scarcity, economic pressure, and increasing reliance on ultraprocessed convenience foods. This phenomenon may be especially consequential in vulnerable populations and helps explain the paradox whereby individuals are expected to manage increasingly complex dietary regimens while lacking practical means to prepare appropriate meals. In this view, cooking-skills education can be framed less as lifestyle enrichment and more as functional rehabilitation supporting health management.

The social environment also operates as a double-edged determinant. Family dynamics are frequently described as a source of unintentional “dietary sabotage” when household preferences and practices conflict with diabetes recommendations [31,77,78]. Similarly, cultural and religious factors, such as Ramadan fasting [70,79,80] or Indigenous traditions [38,81], impose obligations of hospitality that come into direct conflict with medical prescriptions. However, when interventions are culturally competent and involve the family as a unit of care, social support becomes the most powerful facilitator of habit change [52,82].

### Implications for practice and the technological “GAP”

This review highlights a notable technological gap. Although digital tools have expanded rapidly to support individual logistics (recipes, shopping lists, telemedicine), few interventions are designed to strengthen interpersonal relationships and social support (d7) in diabetes care. Many tools remain oriented toward a single-user model, despite the inherently social nature of eating. Although virtual culinary medicine and “tele-cooking” models show promise in reducing access barriers [59,83], there remains a need for a paradigm shift in innovation toward family and community-based approaches. Future technological developments should prioritize tool innovations that connect individuals with families, peers, and community support to foster collective efficacy, rather than focusing predominantly on biometric tracking.

From a clinical perspective, these findings suggest that routine diabetes care should incorporate at least a minimal assessment of domestic food preparation capacity. Simple screening questions regarding cooking confidence, access to cooking facilities, and meal preparation responsibility could help clinicians identify patients at risk of dietary nonadherence despite adequate knowledge. Referral pathways to community-based cooking programs, teaching kitchens, or food prescription initiatives should be considered equivalent in importance to referrals for pharmacological intensification when glycemic targets are not met.

### Limitations and strengths

The findings of this review must be interpreted considering certain methodological limitations inherent to the nature of the available evidence. First, there is notable heterogeneity in study designs, ranging from phenomenological qualitative analyses with small samples (*n* = 7) [38] to population studies based on secondary records [39]. This variability, together with the absence of a standardized (“reference standard”) tool to measure culinary literacy in diabetes, precludes a robust quantitative meta-analysis and limits the direct comparison of intervention effectiveness across studies. Furthermore, although the review includes both pediatric and adult populations, the synthesis does not explicitly differentiate between the 2, which may overlook age-specific developmental and logistical nuances in culinary education. In addition, as is common in scoping reviews, we did not conduct a formal critical appraisal or risk-of-bias assessment of included studies; therefore, our results should be interpreted primarily as an evidence map rather than a definitive evaluation of effectiveness. Moreover, despite no language restrictions, publication bias and database indexing practices may have influenced which studies were retrieved and included, potentially underrepresenting evidence from non-English or nonindexed sources. Second, a large proportion of the included studies were cross-sectional or exploratory [71], which limits the ability to infer causality between improvements in culinary skills (d630) and long-term glycemic control. Consequently, the potential long-term implications and the sustainability of behavior changes identified in these studies remain largely unaddressed. Finally, although the search covered the last decade (2014–2025) to ensure technological relevance, this time restriction may have excluded earlier “classic” educational interventions conducted before the widespread digitalization of health.

Nevertheless, this review presents distinctive strengths that add value to the field. The main contribution is the application of the ICF conceptual framework, which has allowed for the dissection, for the first time, of the functional difference between theoretical self-care (d5) and domestic instrumental competence (d6), offering an analytical clarity often absent in traditional nutritional reviews. Likewise, unlike reviews restricted to the Western clinical setting, this study captures the complexity of the patient’s ecosystem, including evidence on labor dynamics [61], religious celebrations such as Ramadan [70,80], and Indigenous perspectives [38,81]. Finally, the geographical diversity of the sample is highlighted; although 50% of the studies were conducted in the United States, the review incorporates a significant variety of international contexts. We have moved beyond a singular focus on Western clinical settings by including evidence from emerging economies and low-resource environments, such as Brazil, Uganda, and South Africa. Furthermore, the inclusion of studies from European and Asian settings—such as Italy, Poland, Portugal, and Hong Kong—allows for a broader discussion on how different healthcare systems, urban environments, and cultural dietary patterns modulate food literacy. Even within the US-based research, the evidence captures a wide range of ethnic and cultural backgrounds, including veterans and food-insecure populations. By capturing this broad spectrum of structural and cultural landscapes, the review provides a more comprehensive understanding of the global barriers to dietary adherence, which increases the ecological validity of the findings for global public health.

### Recommendations for future research

On the basis of gaps identified in the evidence map, several priorities emerge. First, there is a need to develop and validate psychometric and/or performance-based instruments to measure “culinary competence” and “instrumental self-efficacy” in people with diabetes [55,59]. Future research should move beyond vague self-reported measures in favor of task-based checklists aligned with the ICF d630 domain.

Second, digital interventions should be designed to extend beyond individual logistics and actively support social interaction and shared food practices. Collaborative tools that involve family members or peer networks may help reduce isolation and support sustainable routines, and their effects on diabetes-related distress merit evaluation [42,43]. Third, longitudinal RCTs are needed that integrate teaching-kitchen education with structural components such as food prescription (“Food as Medicine”) initiatives [63,66], assessing biomedical outcomes (e.g., HbA1c) alongside patient-centered outcomes including food security and purchasing behaviors.

Finally, given the influence of culture and identity on food practices [50,63], future research should move beyond simple translation of educational materials toward the codesign of interventions with communities (e.g., adaptations for Ramadan or traditional dietary practices), examining how the cultural competence of educators influences long-term adherence.

In conclusions, this scoping review highlights that much of the diabetes nutrition-education literature and many educational approaches continue to emphasize metabolic targets and knowledge-focused components (i.e., what to eat), whereas comparatively fewer studies and interventions explicitly address the instrumental competencies required to implement recommendations in daily life (i.e., how to cook and manage food-related tasks). Across included studies, nutritional knowledge and diet-management strategies (ICF d1/d5) appear important but may be insufficient for sustained change when practical culinary skills and structural supports (ICF d6) are not considered.

Overall, our mapping suggests that culinary medicine and skills-based approaches may help bridge the gap between dietary advice and real-world implementation. In particular, future educational strategies could consider: *1*) complementing counseling with hands-on training that builds cooking competence and confidence; *2*) integrating culturally responsive adaptations and structural supports that address environmental constraints, especially food insecurity (e.g., Food as Medicine initiatives); and *3*) designing innovations that better reflect the social nature of eating by incorporating family and community support, rather than focusing exclusively on individual tracking.

Ultimately, improving dietary outcomes in diabetes may require not only providing evidence-based recommendations, but also ensuring that individuals are functionally supported to prepare, afford, and share appropriate foods within their everyday environments. Recognizing culinary competence and domestic-life resources as relevant components of diabetes self-management may inform the development of more feasible, equitable, and context-sensitive nutrition education.

## Author contributions

The authors’ responsibilities were as follows – JCM, ES: contributed to conceptualization, methodology, formal analysis, investigation, data curation, writing – original draft preparation, visualization; JCM: contributed to software; MTM-L, SMP, APF, MF, BGT, FEC: contributed to validation, writing—review and editing; SMP, APF, FEC: contributed to resources; ES, SMP: contributed to supervision; ES: contributed to project administration; and all authors: read and agreed to the published version of the manuscript.

## Data availability

The data that support the findings of this study are available on request from the corresponding author, on reasonable request.

## Declaration of generative AI and AI-assisted technologies in the writing process

During the preparation of this manuscript, the authors used DeepL for the purposes of translation of the articles used in this manuscript. The authors have reviewed and edited the output and take full responsibility for the content of this publication.

## Funding

The authors would like to express their sincere gratitude to the Universidad Católica de Valencia San Vicente Mártir for its financial support and for covering the costs associated with the Open Access publication of this article.

## Conflict of interest

The authors report no conflicts of interest.
