# Supplementary material for: Nutritional Needs, Dietary Knowledge, and Culinary Skills in Individuals with Diabetes Mellitus: A Scoping Review
Source: Adv Nutr. 2026 Apr 7;17(5):100627. doi: 10.1016/j.advnut.2026.100627 (PMC13141728; doi:10.1016/j.advnut.2026.100627)
Supplement: Multimedia component 1 [file mmc1.docx]

Nutritional Needs, Dietary Knowledge, and Culinary Skills in Individuals with Diabetes

Mellitus: A Scoping Review

Jorge Casaña Mohedo

**Table S1.** Search strategy

| **Databases** | **Search Date** | **Search string** | **Results** |
| --- | --- | --- | --- |
| Pubmed | 02 Jan 2026 | (((("diabetes mellitus"[MeSH Terms] OR ("diabetes"[All Fields] AND "mellitus"[All Fields]) OR "diabetes mellitus"[All Fields]) AND (("diet"[MeSH Terms] OR "diet"[All Fields]) AND ("food nutr roma"[Journal] OR ("food"[All Fields] AND "and"[All Fields] AND "nutrition"[All Fields]) OR "food and nutrition"[All Fields]))) OR ("nutrition programmes"[All Fields] OR "health planning"[MeSH Terms] OR ("health"[All Fields] AND "planning"[All Fields]) OR "health planning"[All Fields] OR ("nutrition"[All Fields] AND "programs"[All Fields]) OR "nutrition programs"[All Fields])) AND ("feeding behaviour"[All Fields] OR "feeding behavior"[MeSH Terms] OR ("feeding"[All Fields] AND "behavior"[All Fields]) OR "feeding behavior"[All Fields])) AND ((y_10[Filter]))) | 2646 |
| Scopus | 02 Jan 2026 | diabetes mellitus AND Diet, Food AND Nutrition OR Nutrition programs AND Feeding behavior OR cooking | 103 |
| Web of Science | 02 Jan 2026 | (("Diabetes Mellitus" OR "Diabetes Mellitus, Type 2") AND ("Health Education" OR "Nutritional Sciences" OR "Diet, Healthy") AND ("Cooking" OR "Culinary Arts") AND ("Self Care" OR "Self-Management" OR "Patient Compliance")) | 70 |
| Embase | 02 Jan 2026 | ('diabetes mellitus'/exp OR 'diabetes mellitus type 2'/exp) AND ('health education'/exp OR 'nutritional science'/exp OR 'healthy diet'/exp) AND ('cooking'/exp OR 'culinary art'/exp) AND ('self care'/exp OR 'self management'/exp OR 'patient compliance'/exp) | 31 |

*NOTE: Mendeley results refer to catalog search used as supplementary grey literature source.*

**Table S2.** Characteristics of the studies included in the review.

| **Author** | **Country** | **Design** | **Sample (N) & Population** | **Intervention / Phenomenon** | **Culinary Comp.** | **Key Results (KAP)** | **Barriers and Enablers** |
| --- | --- | --- | --- | --- | --- | --- | --- |
| Addala, 2019 | USA | Qualitative (Focus Group) | N=17 (T1D, Young Adults) | Weight management & diet conflicts | N/A | Weight control strategies vs. glycemic control | Barrier: Body image, social pressure. |
| Almansour, 2016 | Australia | Qualitative (Interviews) | N=30 (T2D, Muslims) | Ramadan fasting & diet management | N/A | Habit adjustment during religious fasting | Facilitator: Faith. Barrier: Family feasts. |
| Aparecida, 2020 | Brazil | Observational/Intervention | N/A | Cooking workshops ("Oficinas") | High | Increased fiber intake, Quality of Life | Facilitator: Socialization, learning-by-doing. |
| Araujo, 2023 | Brazil | Methodological | N=31 (19 experts + 12 PHA professionals) | Protocol based on Dietary Guidelines | Low | Content validity of educational material |  |
| Baig, 2015 | USA | Systematic Review | N=26 studies | Family-based interventions | N/A | Impact of family support on self-care | Barrier: Family "sabotage". Facilitator: Support. |
| Bastami, 2023 | Iran | Transversal | N=288 (T2D) | Food Literacy assessment | Low | Higher Food Literacy linked to lower HbA1c | Barrier: Low general education level. |
| Biber, 2023 | USA | Quasi-experimental pilot | N=20 (T2D) | "Food as Medicine" (Edu + Provision) | Medium | Improved self-efficacy, reduced distress | Barrier: Cost (mitigated by intervention). |
| Bowen, 2015 | USA | Controlled Assay (RCT) | N=137 (T2D) | Empowerment vs. Standard Education | Low | Improved self-management & knowledge | Barrier: Low numeracy skills. |
| Brega, 2023 | USA | Validation | N=60 (Indigenous) | Self-efficacy Scales | N/A | Psychometric reliability |  |
| Burner, 2018 | USA | Qualitative | N=24 (T2D, Low-income) | Family role in mHealth interventions | N/A | Family dynamics in digital support | Facilitator: Feeling supported via text. |
| Byrne, 2017 | USA | Retrospective | N=155 (Veterans) | Cooking Classes (Teaching Kitchen) | High | Reduced HbA1c and Blood Pressure | Facilitator: Visual/practical approach |
| Cairns, 2022 | Sweden | Qualitative | N=10 (Dietitians) | Group education in T1D | N/A | Peer learning dynamics | Barrier: Hard to personalize in groups |
| Daramillas, 2025 | Greece | Pre-post Quasi-experimental | N/A | Innovative educational tools | Low | Technology/Maps to improve knowledge | Barrier: Digital divide |
| Dexter, 2019 | USA | Pre-Post Test | N=75 (Veterans) | Cooking Classes (12 weeks) | High | Improved Cooking Confidence |  |
| Goldstein, 2024 | Canada | Mixed/Viability | N=43 (T1D, Children) | "Summerlunch+" (Virtual cooking) | High | High feasibility, improved culinary confidence | Facilitator: Home environment |
| Hawley, 2021 | USA | RCT Protocol | N=165 (Indigenous) | "Cooking for Health" | High | Protocol: Study design | (Results pending) |
| Hempler, 2015 | Denmark | Qualitative | N=12 (Pakistani Immigrants) | Diet education in daily life | N/A | Cultural adaptation of medical advice | Barrier: Hospitality traditions |
| Hung, 2022 | Hong Kong | Mixed | N=185 (T2D, Employees) | Home-cooking for workplace lunch | Medium | Cooking freq. associated with better HbA1c | Barrier: Time, eating-out culture |
| Kiguli, 2019 | Uganda | Qualitative | N=106 (Rural) | Traditional cooking practices | High | Local cooking methods vs. recommendations | Barrier: Fuel/water scarcity, cost |
| Lai, 2024 | Taiwan | Longitudinal | N=Database (Taiwan Share Care) | "Diabetes Share Care" Program | Low | Long-term sustainability of control | Facilitator: Continuity of care |
| Misra, 2025 | USA | Intervention | N=10 (Rural) | Dietary Tracking | Medium | Tracking improves dietary behavior | Barrier: Tracking fatigue |
| Muchiri, 2021 | South Africa | RCT | N=77 (T2D, Low-resource) | Culturally Adapted Nutrition Edu. | Low | Improved self-efficacy & vegetable intake |  |
| Muchiri, 2023 | South Africa | Qualitative | N=45 (T2D) | Experiences in education program | N/A | Perception of habit change | Barrier: Poverty, lack of support |
| Murillo, 2022 | Spain | Narrative Review | N/A | Culinary Strategies (Techniques) | High | Impact of cooking methods on glycemia | Facilitator: Technical culinary knowledge |
| Mutagwanya, 2021 | Uganda | Intervention | N=100 (T2D) | Nutrition Edu. vs Standard Practice | Low | Improved feeding practices |  |
| Panduro, 2024 | Peru | Pre-experimental | N/A | Portion Size Education | Medium | Improved visual knowledge of portions |  |
| Polak, 2018 | USA | Narrative Review | N/A | Home Cooking as intervention | High | Evidence on home cooking impact | Barrier: Time, basic skills |
| Quiñonez, 2019 | México | Qualitative | N=7 (Indigenous) | Subjective bonds with food | N/A | Cultural meaning of food | Barrier: Identity vs. Health conflict |
| Rayala, 2025 | USA | Intervention | N=150 (Underinsured) | Food Skills (Planning, cooking) | High | Reduced stress, improved diet quality | Facilitator: Confidence in management |
| Savarese, 2021 | Italy | Systematic Review | N=33 studies | Food Literacy Interventions | Variable | Patient Engagement | Barrier: Health literacy levels |
| Shapiro, 2024 | USA | Narrative Review | N/A | Cultural Influence on Nutrition | N/A | Importance of cultural competence |  |
| Sharma, 2021 | USA | Quasi-experimental | N=108 (Food Insecurity) | Virtual Culinary Medicine | High | Improved biometrics & shopping habits | Barrier: Internet/food access |
| Short, 2023 | USA | Mixed/Viability | N=21 (Food Insecurity) | Food-based education | Medium | High acceptability/feasibility |  |
| Short, 2023 | USA | Protocol | N/A | "Delivering Health" (Food boxes) | Medium | Protocol: Study design | (Results pending) |
| Short, 2024 | USA | RCT Protocol | N/A | Food Delivery + Education | Medium | Protocol: Study design | (Results pending) |
| Shrodes, 2021 | USA | RCT Pilot | N=13 (T1D/T2D) | "Cooking Matters" (Feasibility) | High | Feasibility of cooking classes | Barrier: Attendance/Transport |
| Sinska, 2022 | Poland | Transversal | N=394 (T1D) | Nutritional behaviors | Low | Frequency of self-care habits |  |
| Stortz, 2021 | EE. USA. | Qualitative | N=9 experts | Indigenous perspectives on curriculum | N/A | Curriculum adaptation needs | Barrier: Historical trauma |
| Tete 2021 | Brazil | Qualitative | N=10 (T2D) | Food & Nutrition Education | Low | Perception of knowledge acquisition | Barrier: Difficulty changing habits |
| Tripathi, 2023 | India | Qualitative | N=27 (Patients+Prov) | Dietary Barriers & Facilitators | N/A | Dual perspective (Patient vs Doctor) | Barrier: Family preferences, cost |
| Uliana 2022 | Brazil | Transversal | N=472 (T1D) | Carb Counting Adherence | High | Technique adherence linked to diet quality | Barrier: Mathematical complexity |
| Vasconcelos 2021 | Portugal | RCT Pilot | N=33 (T2D) | Community Food Education | Low | Improved dietary pattern |  |
| Venkatesh 2024 | USA | Intervention | N=1574 adults | "Cooking Well with Diabetes" | High | Healthy cooking practices |  |
| Weller 2021 | USA | Transversal | N=465 adults | Sustainable lifestyle strategies | N/A | Long-term maintenance strategies | Facilitator: Sustainability |
| Wetherill, 2019 | USA | Transversal | N=16826 (Food Pantry) | Coping Strategies | N/A | Negative coping (diluting food) | Barrier: Severe food insecurity |
| Williams, 2023 | USA | RCT | N=48 (T1D/T2D) | "Cooking Matters" | High | Improved HbA1c & Self-efficacy | Facilitator: Free food provision |
